# Gender Differences in the Motivations for Suicide Attempts Among Cases Presenting to the Emergency Department: A Retrospective Study

**DOI:** 10.1192/j.eurpsy.2026.11981

**Published:** 2026-07-31

**Authors:** H. Ulusoy, T. Demircan, K. Karbeyaz, M. E. Çanakçı, I. G. Yılmaz Karaman

**Affiliations:** 1 Department of Psychiatry; 2 Department of Forensic Medicine; 3 Deparment of Emergency Medicine, Eskişehir Osmangazi University Faculty of Medicine, Eskişehir, Türkiye

## Abstract

**Introduction:**

Approximately 740,000 people die by suicide each year. A previous suicide attempt is one of the strongest predictors of death by suicide. Identifying the factors that trigger suicidal behavior can provide opportunities for protective and preventive interventions. Gender, as a determinant of health, may affect women and men differently and influence the reasons for suicide attempts.

**Objectives:**

The aim of this study is to examine and compare the reasons for suicide attempts among women and men who presented to the emergency department.

**Methods:**

In this study, psychiatric consultations requested for patients who presented to the emergency department due to a suicide attempt or preparatory actions in 2023 were retrospectively reviewed.

**Results:**

Of a total of 746 emergency psychiatric consultations, 110 were assessed as suicide attempts, and 9 as preparatory suicidal actions. The most frequently reported reason for suicide in both genders was domestic relationship problems (51.3%). Men reported economic difficulties and unemployment as reasons for suicide significantly more often than women (p<0.001).

**Conclusions:**

This study highlights the importance of gender roles and family relationships in understanding suicidal behavior. Psychiatric practice should adopt holistic and gender-sensitive approaches, particularly considering issues such as unemployment among men. Preventive strategies should promote healthy interpersonal relationships due to their protective impact against suicidal behavior.

**Disclosure of Interest:**

None Declared

